# Spinal manual therapy in infants, children and adolescents: A systematic review and meta-analysis on treatment indication, technique and outcomes

**DOI:** 10.1371/journal.pone.0218940

**Published:** 2019-06-25

**Authors:** Femke Driehuis, Thomas J. Hoogeboom, Maria W. G. Nijhuis-van der Sanden, Rob A. de Bie, J. Bart Staal

**Affiliations:** 1 IQ Healthcare, Radboud Institute for Health Sciences, Radboud University Medical Center, Nijmegen, the Netherlands; 2 Caphri Research School, Department of Epidemiology, Maastricht University, Maastricht, the Netherlands; 3 Research Group Musculoskeletal Rehabilitation, HAN University of Applied Sciences, Nijmegen, the Netherlands; All India Institute of Medical Sciences, Bhopal, INDIA

## Abstract

**Background:**

Studies on effectiveness and safety of specific spinal manual therapy (SMT) techniques in children, which distinguish between age groups, are lacking.

**Objective:**

To conduct a systematic review of the evidence for effectiveness and harms of specific SMT techniques for infants, children and adolescents.

**Methods:**

PubMed, Index to Chiropractic Literature, Embase, CINAHL and Cochrane Library were searched up to December 2017. Controlled studies, describing primary SMT treatment in infants (<1 year) and children/adolescents (1–18 years), were included to determine effectiveness. Controlled and observational studies and case reports were included to examine harms. One author screened titles and abstracts and two authors independently screened the full text of potentially eligible studies for inclusion. Two authors assessed risk of bias of included studies and quality of the body of evidence using the GRADE methodology. Data were described according to PRISMA guidelines and CONSORT and TIDieR checklists. If appropriate, random-effects meta-analysis was performed.

**Results:**

Of the 1,236 identified studies, 26 studies were eligible. Infants and children/adolescents were treated for various (non-)musculoskeletal indications, hypothesized to be related to spinal joint dysfunction. Studies examining the same population, indication and treatment comparison were scarce. Due to very low quality evidence, it is uncertain whether gentle, low-velocity mobilizations reduce complaints in infants with colic or torticollis, and whether high-velocity, low-amplitude manipulations reduce complaints in children/adolescents with autism, asthma, nocturnal enuresis, headache or idiopathic scoliosis. Five case reports described severe harms after HVLA manipulations in four infants and one child. Mild, transient harms were reported after gentle spinal mobilizations in infants and children, and could be interpreted as side effect of treatment.

**Conclusions:**

Based on GRADE methodology, we found the evidence was of very low quality; this prevented us from drawing conclusions about the effectiveness of specific SMT techniques in infants, children and adolescents. Outcomes in the included studies were mostly parent or patient-reported; studies did not report on intermediate outcomes to assess the effectiveness of SMT techniques in relation to the hypothesized spinal dysfunction. Severe harms were relatively scarce, poorly described and likely to be associated with underlying missed pathology. Gentle, low-velocity spinal mobilizations seem to be a safe treatment technique in infants, children and adolescents. We encourage future research to describe effectiveness and safety of specific SMT techniques instead of SMT as a general treatment approach.

## Introduction

Is manual therapy effective in reducing or resolving complaints or symptoms in infants, children or adolescents? Is it a safe therapeutic approach? Which specific manipulative techniques are performed? In the field of pediatric care, these questions raise interest of healthcare professionals, parents and other stakeholders. Worldwide, manual therapy is performed in infants (<1 year), children (1–11 years) and adolescents (12–18 years), by various healthcare professionals with different therapeutic backgrounds.[1, 2] They use different conceptual frameworks regarding the relationship between symptoms and underlying spinal dysfunction. Manipulative therapeutic techniques differ between professionals and health conditions, and between infants and children/adolescents.[3–7] Distinctions in techniques are made between high-velocity, low-amplitude (HVLA) manipulations[8] and low-velocity mobilizations which can be performed to the full spine or to specific spinal segments. Moreover, treatment indications vary extensively. Infants and children are frequently treated for musculoskeletal conditions, such as movement related complaints,[9] or non-musculoskeletal conditions, including colic, otitis media and asthma.[1, 4, 10] Adolescents are mainly treated for musculoskeletal conditions, such as scoliosis and headache.[1, 2, 4, 10] Non-musculoskeletal conditions as treatment indication in children differs from manipulative treatment approaches in adults, which are mainly focused on musculoskeletal conditions, such as headache, neck pain and low back pain.[11–16]

Pediatric manual therapy and its safety has provoked debates and ethical challenges.[17–19] Although several literature reviews summarize the evidence of manual therapy in children with various indications,[2, 4, 5] systematic reviews describing effectiveness of specific manual therapeutic treatment techniques, specified by treatment indication and age group, are lacking, especially in the field of spinal manual therapy (SMT).[14] Hypotheses regarding underlying spinal dysfunction that could be related to complaints in children differ between professionals, and the therapeutic approaches used within SMT overlap. This overlap impedes the interpretation of effects and harms of SMT. In addition, research concludes on SMT as a general treatment approach instead of on the used techniques. A clear overview of the current state of the evidence is therefore needed to assess the value of specific SMT techniques in different age groups.[20, 21] This systematic review and meta-analysis of the literature provides a broad overview of the evidence regarding the effectiveness and harms of specific SMT techniques in infants, children and adolescents, related to specified treatment indication.

## Methods

We report the results of our systematic review in accordance with the PRISMA guidelines.[22] Prior to the study, the review protocol was registered at PROSPERO (CRD42017056031).

### Literature search strategy

**Fig 1:**
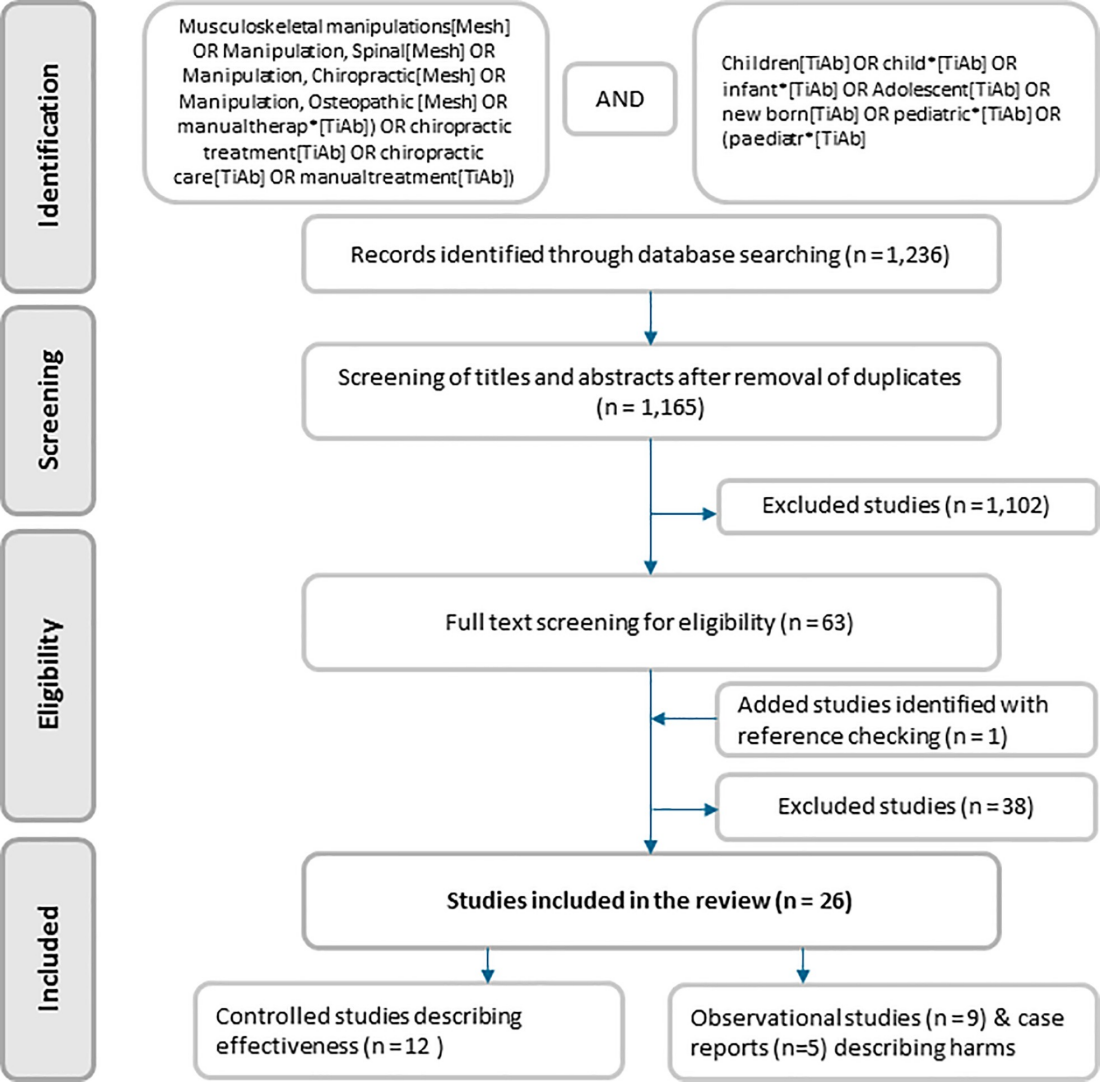
Flowchart search strategy.

The following electronic databases were searched up to 20 December 2017: PubMed, Index to Chiropractic Literature, Embase, CINAHL and Cochrane Library. The scientific literature was systematically searched, combining key words related to “manual therapy” and key words related to “children”. The search strategy for PubMed is shown in Fig 1. The searches in other databases were consistent with this strategy. Reference checking of included articles was used to identify potential studies that were missed with the initial search strategy (n = 1).

### Definitions

To date, there is no international consensus on the specific definition of manual therapy in pediatrics. Overall, three different therapeutic approaches can be recognized. First, chiropractic manual therapy, which uses high-velocity spinal manipulation or instrumented adjustments using minimal forces (e.g. using an Activator).[1, 23, 24] It aims to influence the nervous system, visceral functions and/or soft tissue tensions to correct segmental joint dysfunction.[18, 25, 26] Besides spinal manipulative therapy, chiropractic manual therapy incorporates additional therapies, such as soft tissue massage, nutritional counseling and exercise.[27] Second, osteopathic manual therapy, which follows a similar line of reasoning, but also intends to maintain or restore the flow of body fluids and to support homeostasis of the body.[26, 28] Third, spinal manual therapy (SMT), which relies on segmental, single spinal joint low-force oscillating mobilizations and HVLA manipulations,[8] focuses on the biomechanical aspect of spinal dysfunction by eliciting neurological, physiological and/or muscular changes.[29]

SMT techniques are integrated in all these treatment approaches, but conclusions on effectiveness and safety are mainly given on treatment approach instead of treatment technique. Hence, in this systematic review we focused on specific treatment techniques instead of SMT as a general treatment approach.

In our systematic review, manual therapeutic interventions in which treatment techniques were primarily performed on the full spine or on specific spinal segments, by any healthcare professional, were indicated as SMT. We made a distinction between two main SMT techniques: manipulation and mobilization. Manipulation was described as a HVLA or low-velocity thrust, resulting in a mechanical response of articular surface separation and a cracking sound, which is also defined as cavitation in the affected joint.[8] Mobilization was described as low-velocity, low-amplitude oscillating spinal joint play, without a thrust and without cavitation. Infants were defined as those aged between 0 to 12 months; children were defined being between 1 and 11 years; adolescents as being between 12 and 18 years. Treatment indications were categorized as musculoskeletal or non-musculoskeletal conditions. Hypothesized dysfunction could be postulated to have had a primarily biomechanical, neuroreflectory or physiological origin in the spine or could be described as dysfunction of the whole body, such as disturbed flow of body fluids, myofascial, visceral or parietal bone problems. Treatment outcomes were defined as patient- or parent-reported outcomes, such as symptoms (e.g. asymmetry), behavior (e.g. crying), perceived effect, and quality of life and/or as intermediate outcomes, which were related to therapist-reported impairment or function, such as asymmetry, spinal mobility, spinal dysfunction, or performance. Harms were also interpreted as a treatment outcome and were classified as; mild (transient side effect, lasting <24 hours), moderate (requiring medical and/or general practitioner treatment) and severe (requiring hospital treatment or adverse event; life threatening situation or death).[30]

### Selection procedure and criteria for eligibility

The initial search was performed by the primary author (FD). All studies were collected using EndNote, an online library system, which enabled us to remove duplicates. Screening of titles and abstracts was performed by one author (FD) using predefined eligibility criteria (S1 Table). Controlled studies were included to investigate effectiveness and harms. Observational studies and case reports were included to investigate harms.[31, 32] Subsequently, two authors (FD, TH) independently reviewed the full text of potentially relevant articles for eligibility. Discrepancies were discussed with all authors until consensus was reached, and eligible studies were included for an in-depth review.

### Assessment of risk of bias of individual studies

The assessment of risk of bias was done independently by two authors. Risk of bias of controlled studies was assessed using the Cochrane Risk of Bias tool, focusing on selection-, performance-, detection-, attrition- and reporting bias[33] by FD and JBS. Observational studies were assessed with the Item Bank for Assessing Risk of Bias and Confounding for Observational Studies of Interventions or Exposures (RTI Item Bank)[34] by FD and TH, focusing on selection-, performance-, detection-, attrition- and reporting bias, and confounding. Risk of bias of case reports was assessed using the JBI Critical Appraisal Checklist for Case Reports[35] by FD and JBS.

### Data extraction and analysis

Data extraction was performed by FD using a Summary of Findings table, and thereafter checked by TH in a random sample of 8 studies. Outcomes of effectiveness and harms were described separately. The CONSORT checklist[36] in conjunction with the TIDieR checklist[37] were used to describe the extracted data from controlled studies focusing on study population, treatment indication, hypothesized dysfunction, specific SMT treatment technique and outcomes. If appropriate, study outcomes were pooled. For random effects meta-analysis, outcomes of controlled studies were transformed to standardized mean differences between baseline and follow-up according to Cochrane recommendations.[33] Meta-analysis was performed when two or more studies described a similar intervention and comparable control treatment, and used a similar study population regarding condition and age. If appropriate, intervention groups (≥2 groups) were combined into a single group according to the Cochrane Handbook. Statistical heterogeneity of the intervention effect was assessed using the I^2^ statistic (>50% indicates high heterogeneity).[33] All analyses were conducted using Stata Software, version 12.0 (Stata Inc., College Station, Texas). If studies were not similar, meta-analysis was not considered appropriate, and findings were narratively reported. Data extraction to describe harms detailed treatment indication, specific SMT treatment technique and the reported harm.

### Assessment of quality of body of evidence

Quality of the body of evidence related to effectiveness was assessed using the Grading Recommendations Assessment, Development and Evaluation (GRADE) criteria.[38, 39] Each outcome was assessed in the previously specified age group and treatment indication using five criteria: 1) risk of bias,[40] 2) inconsistency,[41] 3) indirectness,[42] 4) imprecision[43] and 5) publication bias.[44] The assessment using GRADE was based on data from the assessment of risk of bias and the data extraction process. The completion of the GRADE tables was done by FD. The quality of the body of evidence was assigned as high, moderate, low or very low (Box 1) and described according to Cochrane recommendations.[45] Randomized controlled studies were considered high quality evidence and were downgraded by one level for serious concerns and by two levels for very serious concerns.[31, 46] Non-randomized controlled studies were automatically downgraded for limitations in the study design. They were further downgraded for any concerns in the five grading criteria. If the number of studies per specific age group, intervention and outcome was limited, inconsistency could not be graded and was interpreted as ‘unknown’.[47] For each comparison and outcome measure, a GRADE table was completed. Because of the varying designs of studies that solely described harms of SMT, GRADE was not used; instead, results were reported narratively.

**Box 1. GRADE levels describing the quality of the body of evidence (39)**

GRADE levels

***High*:** Research provides a very good indication of the likely effect. The likelihood that the effect will be substantially different is low.

***Moderate*:** Research provides a good indication of the likely effect. The likelihood that the effect will be substantially different is moderate.

***Low*:** Research provides some indication of the likely effect. The likelihood that the effect will be substantially different is high.

***Very low*:** Research does not provide a reliable indication of the likely effect. The likelihood that the effect will be substantially different is very high.

## Results

Electronic database searching identified 1,236 articles. After removing duplicates, 1,165 records were screened on title and abstract. A total of 1,102 records were excluded because of ineligible intervention, study design or study population. For the remaining 63 articles, eligibility was assessed based on full-text; 38 were excluded because of study population (n = 5), study design (n = 17), outcomes (n = 8) or the intervention could not be described as SMT (n = 8) (S2 Table); reference checking added one study (Fig 1). In total 26 studies were included; 12 controlled trials, of which 10 were randomized controlled trials,[48–59] 9 observational studies[60–68], and 5 case reports.[69–73]

Methodological limitations of controlled studies were related to unclear allocation concealment, partial or no blinding of participants and personnel, and incomplete outcome reporting. Limitations of observational studies were related to performance, detection and attrition bias, and selective outcome reporting. Limitations of case reports were lack of detail or unclear description of the intervention or treatment procedure. Outcomes of the quality assessments are presented in S3 and S4 Tables.

### Effectiveness

Study characteristics on treatment indication, hypothesized dysfunction, treatment technique and outcomes of the included 12 controlled studies are shown in Table 1. In the studies involving infants (n = 5), interventions consisted of low-force, gentle, light fingertip spinal mobilizations. In studies involving children/adolescents (n = 7), HVLA thrust spinal manipulations were most frequently used (n = 6). Control interventions consisted of no treatment (n = 3), sham treatment (n = 4) or other treatments (n = 5), such as physical therapy, medication and manual therapy using the drop mechanism (Table 1).

**Table 1:** Treatment indication, hypothesized dysfunction, treatment technique, outcome measures and outcomes of controlled studies (n = 12) on effectiveness of SMT in infants, children and adolescents.

**Studies involving infants**									
**Treatment indication**	**Authors**	**Study population (age)**	**Hypothesized dysfunction**	**Intervention** **(IV)**	**Outcome measures**	**Comparator** **(C)**	**Outcomes**	**Risk of** bias*	GRADE**
Colic (N-MSK)	Olafsdottir et al., 2001 [49]	86 infants (3–9 weeks)	Spinal joint dysfunction	Spinal mobilizations using light fingertip pressure, performed by a chiropractor	Crying hours/day after 8 days	No treatment (infants were just held)	Both groups decreased crying hours/day (IV: -2 (SD:2.1), C: -2.3 (SD: 2.2)). No significant difference between groups (p:0.37).	Moderate	Very low quality of evidence
Colic (N-MSK)	Miller et al., 2012 [50]	104 infants (<8 weeks)	Not described	Spinal low-force mobilizations (1 blinded group (IV), 1 not-blinded group (IV-nb)), performed by a chiropractor	Crying hours/day after 10 days	No treatment (infants were not touched)	Both groups decreased crying hours/day (IV: -2.4 (SD:2.5), IV-nb: -2.8 (SD:2.2), C: -1.0 (SD:1.6)). Significant (*p*<0.05) decrease (-1.4) in IV group compared to no treatment.	Moderate	
Colic (N-MSK)	Browning & Miller, 2008 [48]	43 infants (<8 weeks)	Not described	Spinal low-force mobilizations, performed by a chiropractor	Crying hours/day after 14 days	Occipito-sacral decompression	Both groups decreased crying hours/day (IV: -2.1 (SD:2.2), C: -2.0 (SD:1.4)). No significant difference between groups (p:0.85).	Moderate	Very low quality of evidence
Colic (N-MSK)	Wiberg et al., 1999 [51]	50 infants (2–10 weeks)	Spinal joint dysfunction	Spinal mobilizations using light fingertip pressure, performed by a chiropractor	Crying hours/day after 14 days	Dimethicone medication	Both groups decreased crying hours/day (IV: -2.4 (SD:0.4), C: -1.0 (SD:0.6)). Significant decrease of crying hours (-1.7 hours/day) in IV group compared to medication (*p* = 0.04).	High	
Torticollis (MSK)	Haugen et al., 2010 [52]	32 infants (3–6 months)	Upper cervical dysfunction	Spinal low-force mobilizations by a manual therapist and pediatric physical therapy	Change in torticollis after 8 weeks	Pediatric physical therapy	In both groups torticollis positively changed (IV: 80% improvement, C: 81.3%). No significant difference between groups (p:0.85).	Moderate	Very low quality of evidence
**Studies involving children and/or adolescents**									
**Treatment indication**	**Authors**	**Study population (age)**	**Hypothesized dysfunction**	**Intervention** **(IV)**	**Outcome measures**	**Comparator** **(C)**	**Outcomes**	Risk of bias*	GRADE**
Asthma (N-MSK)	Balon et al., 1998 [53]	91 children (7–16 years)	Spinal joint dysfunction	Spinal HVLA manipulations, performed by a chiropractor	Peakflow (FEV_1_), symptoms, medication use and quality of life after 16 weeks	Low-velocity, low-amplitude push in gluteal and scapulae region	Both groups showed small increases in peakflow (IV: 103.6% (SD:13.7), C: 104.3% (SD:13.3)), improvement in symptoms and quality of life and decrease in medication use. No significant differences between groups (p:0.82).	High	Very low quality of evidence
Asthma (N-MSK)	Bronfort et al., 2001 [54]	36 children (6–17 years)	Spinal joint dysfunction	Spinal HVLA manipulations, performed by a chiropractor, and standard medical treatment	Peakflow (FEV_1_), medication use and quality of life after 12 weeks	Light gentle spinal pressure, without a thrust, standard medical treatment	Little insignificant increase in peakflow and quality of life and decrease in medication use in intervention group. Control group outcomes not reported. Groups could not be compared.	NA	
Autism (N-MSK)	Khorsid et al., 2006 [56]	14 children (age not specified)	Not described	Upper cervical manipulations, using the Atlas Orthogonal, performed by a chiropractor	Autism related symptoms after 3 months	Diversified technique SMT on the full spine	Both groups decreased in symptoms (IV: -32%, C:-19%). No significant difference between groups (p-value not reported).	High	Very low quality of evidence
Headache (MSK)	Borusiak et al., 2009 [55]	56 children (7–15 years)	Cervical joint dysfunction	Cervical HVLA manipulation, performed by a manual therapist	Headache duration (hours) and intensity (VAS scale) after 2 months	Light touch of spinal segments	Both groups decreased in symptoms (duration IV:-7.5, C:-6.6; intensity IV:-0.3, C:0.1). No significant differences between groups (p>0.05).	Moderate	Very low quality of evidence
Nocturnal enuresis (N-MSK)	Reed et al., 1994 [57]	46 children (5–13 years)	Spinal joint dysfunction	HVLA manipulations, performed by a chiropractor	Frequency of bed wetting after 12 weeks	Instrumented adjustment using an Activator on the thoracic area	Intervention group decreased in frequency (IV:-1.2% (SD:2.2), C:+17.9% (SD:46.1%). No significant difference between groups (p:0.07).	Moderate	Very low quality of evidence
Idiopathic scoliosis (MSK)	Swierkosz & Nowak, 2015 [58]	35 adolescents (15–18 years)	Spinal joint dysfunction	Lower lumbar segmental mobilizations and traction, performed by a physical therapist	Back pain and quality of life after 3 weeks	No treatment	Pain decreased and physical health related quality of life increased (*p*<0.001) within IV group. No between group comparisons were reported.	NA	Very low quality of evidence
Grip strength-ening (MSK)	Botelho & Andrade, 2012 [59]	18 judo athletes	Stimulate nerve innervations	Cervical HVLA manipulations, performed by a chiropractor	Grip strength	SMT using the head piece drop mechanism	Significantly better grip strength (*p*<0.05) in IV (+13.7% mean left/right hand) compared to C (+5%) (p:0.0025).	Moderate	Very low quality of evidence

IV: Intervention group, C: Control group, MSK: musculoskeletal, N-MSK: non-musculoskeletal, SMT: spinal manual therapy, HVLA: high-velocity, low-amplitude, FEV_1_: forced expiratory volume at the end of the first second of expiration

* Risk of bias table is shown in Table A in S3 Table

** Detailed information about the GRADE assessment (GRADE tables) are presented in S4 Table

#### Effectiveness of SMT techniques in infants

The review included five studies evaluating SMT techniques in infants. Four studies included infants with colic [48–51] and one study infants with torticollis.[52] Outcomes are presented in Table 1.

**Infants with colic**

Two studies compared SMT to no treatment.[49, 50] Miller et al. compared a blinded treatment group (n = 35), non-blinded treatment group (n = 33) and a non-treatment group (n = 34) and found that crying hours significantly decreased (*p*<0.05) with 1.5 hours/day after 10 days between blinded treatment and non-treatment.[50] Olafsdottir et al. showed no significant differences between the SMT (n = 46) and control group (n = 34) in decrease of crying hours/day (-2 and -2.3, respectively) after 8 days.[49] Before meta-analysis, the two intervention groups of Miller et al. were combined into one single intervention group. Analysis of the overall pooled effect of SMT versus no treatment on crying hours/day was -0.33 (95% CI: -0.12 to 0.59; *I*^*2*^: 89.1%, *p*:0.484). Two studies compared SMT to other treatments.[48, 51] Browning & Miller found a decrease in crying hours/day of 2.1 hours after SMT (n = 22) and 2.0 hours after occipitosacral decompression (n = 21) 14 days post-treatment. Groups differed not significantly.[48] Wiberg et al. compared SMT (n = 25) to daily dimethicone medication (n = 25) and found a significant decrease in crying hours/day in favor of the SMT group (-2.4 vs. -1.0, *p* = 0.04).[51] No meta-analysis could be performed, due to incomparability of the control treatments. Because of very low quality evidence (serious risk of bias, very serious inconsistency, serious indirectness, serious imprecision) we are uncertain whether SMT consisting of spinal mobilizations reduces crying hours/day in infants with colic.

**Infants with torticollis**

Haugen et al. compared pediatric physical therapy combined with SMT (n = 16) to pediatric physical therapy alone (n = 16) on change in torticollis and cervical mobility, and found no significant differences (SMT improved 80%, pediatric physical therapy alone improved 81.3%).[52] Because of very low quality evidence (unknown inconsistency, very serious imprecision) we are uncertain about the effect of SMT consisting of spinal mobilizations on change of torticollis and increased cervical mobility in infants.

#### Effectiveness of SMT techniques in children/adolescents

Seven studies investigated the effectiveness of SMT in children and/or adolescents (Table 1).[53–59]

**Children/adolescents with asthma**

Two studies compared SMT to sham treatment on lung function and asthma related symptoms in children.[53, 54] Balon et al. compared spinal HVLA manipulation (n = 38) to sham treatment with low-velocity, low-amplitude push in the gluteal and scapulae region (n = 42). After 16 weeks, lung function (+103.6% after SMT vs. +104.3% after sham treatment), quality of life and reduction in medication were not significantly different between groups.[53] Bronfort et al. compared HVLA spinal manipulations (n = 24) to light gentle manual pressure (sham treatment) to the spine (n = 12), and found no significant difference between groups in lung function, quality of life and medication use.[54] No meta-analysis could be performed, because Bronfort et al. only reported data of outcomes of the intervention group. We contacted the author, but did not get a response. Because of very low quality evidence (serious risk of bias, serious inconsistency, very serious imprecision) we are uncertain whether SMT consisting of HVLA manipulations improves lung function in children/adolescents with asthma.

**Children/adolescents with autism**

Khorshid et al. compared upper cervical SMT (n = 7) to full spine diversified care (n = 7) on autism related symptoms. No significant differences between groups were found (32% improvement after SMT, 19% after diversified care).[56] Because of very low quality evidence (serious risk of bias, unknown inconsistency, very serious imprecision) there is uncertainty about the effect of SMT consisting of upper cervical manipulations on reducing autism related symptoms in children/adolescents with autism.

**Children/adolescents with headache**

Borusiak et al. compared cervical HVLA manipulation (n = 28) to light touch of spinal segments as sham treatment (n = 28) on headache related symptoms (e.g. days with headache, duration, intensity) and showed no significant differences after 2 months.[55] Outcomes of HVLA manipulation versus sham treatment were; days with headache -9.7% vs. -9.4%, duration (hours) -7.5% vs. -6.6%, intensity (VAS scale) -0.3 vs. 0.1. Because of very low quality evidence (unknown inconsistency, very serious imprecision) we are uncertain about the effect of cervical SMT with HVLA manipulations on reducing headache related symptoms in children/adolescents with headache.

**Children/adolescents with nocturnal enuresis**

Reed et al. compared HVLA adjustments (n = 31) to sham treatment using an Activator at a non-tension area in the thoracic spine (n = 15). There were no significant differences between groups after 12 weeks in the frequency of bed-wetting (-1.2% after HVLA adjustments, +17.9% after Activator).[57] Because of very low quality evidence (serious risk of bias, unknown inconsistency, very serious imprecision) we are uncertain whether SMT consisting of HVLA manipulations reduces the frequency of bed-wetting in children with nocturnal enuresis.

**Children/adolescents with idiopathic scoliosis**

Swierkosz & Nowak compared segmental spinal mobilizations and traction at level L5-S1 (n = 21) to no treatment (n = 11) on back pain and quality of life. Post-treatment outcomes were only reported for the SMT group. Hence, between group comparison were not described.[58] Because of very low quality evidence (serious risk of bias, unknown inconsistency, very serious imprecision) there is uncertainty about the effect of segmental spinal mobilizations on reducing back pain and increasing quality of life in adolescents with idiopathic scoliosis.

**Healthy adolescent judo athletes**

Botelho & Andrade compared cervical HVLA manipulations (n = 9) to adjustments using the head piece drop mechanism (n = 9) on grip strength immediately after treatment. After cervical HVLA manipulations adolescents showed significantly (*p*:0.0025) better grip strength in both hands (mean increase 13.7%) compared to the control group (+5%).[59] Because of very low quality evidence (unknown inconsistency, serious risk of bias, very serious imprecision) we are uncertain whether cervical HVLA SMT increases transient grip strength in healthy adolescents.

### Harms

Nine observational studies[60–64, 66–68, 74], five case reports[69–73], and four controlled studies[50, 53, 55, 59] reported on harms. Patient characteristics, treatment indication, treatment technique and related harms are shown in Table 2.

**Table 2:** Studies on harms of spinal manual therapy: Patients, treatment indication and treatment technique.

Study population	Treatment indication	Clinical history	Reported harm	Treatment technique	Study design	Author	Risk of bias
**Cervical spinal manipulation in infants**							
4 month old boy	Congenital torticollis	A few hours after manipulation, the infant was difficult to arouse, was limp, pale and moaning. Infant’s mother went back to the chiropractor, who manipulated the neck again. Thereafter the infant moaned and grunted continuously. Three hours after the second cervical manipulation, the infant was hospitalized, had a seizure and was comatose. He suffered from paralysis of both legs and the right arm. MRI showed a spinal cord tumor, which was immediately removed. After surgery, motor and sensory function regained to T4-level. 18 months postoperatively, the child had full use of his arms, sensory function at T9-level and some spontaneous but nonfunctional motion of the right leg.	Temporary quadriplegia	Cervical spinal manipulation towards flexion- extension and axial (un)loading, performed by a chiropractor	Case report	Shafrir & Kaufman, 1996 [70]	Moderate
3 month old girl	Minimal motor restlessness	After manipulations, the infant cried heavily and developed fecal incontinence and breathed loudly. After 10 minutes infant’s lips turned blue, muscles were weak and there was no response on touching. Infant’s father started CPR until ambulance took over. After 1 hour, infant had her own heart rhythm again. After hospital exam no abnormalities were found on x-ray and CT. MRI showed abnormalities in the pons and mesencephalon confirming vertebrobasilar ischemia, specifically in the spinal cord. 12 hours after manipulation treatment, infant had no spontaneous breathing, brainstem reflexes and tendon reflexes. Hospital treatment was stopped and infant died within minutes. Autopsy showed infarcts in spleen and heart due to oxygen deficiency and multi organ failure.	Death	Manipulations of the (cervical) spine towards forced full spine flexion, performed by a cranio-sacral therapist	Case report	Holla et al., 2009 [71]	High
3 month old girl	Torticollis and muscular hypotonic	Ten minutes after treatment, the infant looked pale and had blue lips, cold legs, blue/black skin and breathing difficulties. Infant was hospitalized because of asystole. CPR was started and the heart was defibrillated for 25 minutes. The infant suffered from bleeding into the vertebral arteries at C1 resulting in caudal brainstem ischemia and subarachnoid hemorrhage. Authors state that underlying cardiovascular and neurological issues before starting the treatment could not be ruled out.	Death	Cervical spinal manipulation towards forced rotation according to the Vojta method, performed by a physical therapist	Case report	Jacobi et al., 2001 [72]	Low
**Cervical spinal manipulation in children/adolescents**							
6 year old boy	Sinus infection	The day after manipulation, child experienced complaints of tingling and numbness in the left arm and developed gradual weakness of the left arm during the week. Two weeks after manipulation MRI showed a bilateral lesion in the ventral horns of the spinal cord from C3 –C7. A vascular compromise of vertebral arteries resulting in anterior cordischemia was proposed.	Muscle weakness in the arm	Cervical spinal manipulation, performed by a chiropractor	Case report	Deputy, 2004 [69]	Moderate
Cohort of 52 children	Headache	Children were randomized to SMT or sham treatment. Evaluation of side effects was performed immediately after treatment and after the 2-month follow-up period.	Mild harms: dizziness (n = 11), hot skin (n = 26)	Cervical HVLA manipulation, performed by a manual therapist	RCT	Borusiak et al., 2009 [55]	Moderate
Cohort of 18 adolescent judo athletes	Grip strength improvement	Adolescents were randomized to SMT or sham treatment. Side effects were evaluated during and after treatment.	Mild harms: neck pain (n = 1), headache (n = 1)	Cervical manipulation consistent with the Diversified technique, performed by a chiropractor	RCT	Botelho & Andrade, 2012 [59]	Moderate
**Full spine manipulation in infants**							
No studies							
**Full spine manipulation in children/adolescents**							
Cohort of 171 children	Nocturnal enuresis	Children were treated and outcomes were monitored and reported by their parents.	Mild harms (pain, headache, stiffness, n = 2)	Chiropractic adjustments on the area of dysfunction, performed by chiropractors	Pros-pective cohort	LeBoeuf et al., 1991 [64]	Moderate
Cohort of 54 pediatric patients	Low back pain	Abstraction from records of included consecutive pediatric patients.	No harms	Lumbar spinal manipulation, performed by chiropractors	Pros-pective cohort	Hayden et al., 2003 [63]	Moderate
Cohort of 577 cases of children (0–18 years)	Various conditions	A survey was used to describe pediatric chiropractic practice, including safety. 21 chiropractors reported on 577 cases in which children (0–18 years) received SMT, in a total of 5,438 visits. Parents reported on 239 children after treatment. Chiropractors and patients or parents documented treatment-associated changes, such as aggravations (worsening or complaints), complications or improvements.	Mild harms: stiffness, soreness (n = 3)	Various techniques, e.g. diversified-, Gonstaed-, Thompson- and cranial technique, performed by chiropractors	Cross-sectional study	Alcantara et al., 2009 [60]	Moderate
Cohort of 781 cases of pediatric patients (<3 years)	Various conditions	Pediatric case files were checked to identify any adverse effects after chiropractic care.	Mild harms: crying (n = 4), restlessness, not feeding well, head tilt.	Various techniques, e.g. full spine manipulation, cervical manipulation, occipital-sacral decompression, performed by chiropractors	Retro-spective review	Miller & Benfield, 2008 [67]	Moderate
Cohort of 91 children	Asthma	Children were randomized to SMT or sham treatment. Side effects were evaluated using completed diaries.	No harms	Spinal HVLA manipulation, performed by a chiropractor	RCT	Balon et al., 1998 [53]	High
**Cervical mobilizations in infants**							
Cohort of 695 infants	Upper cervical dysfunction and asymmetry	Heart rate, blood pressure, breathing frequency, oxygen saturation and peripheral temperature before, during and after the application of a high cervical impulse were compared. In 47% a change in heart rate was noticed. In 40%, heart rate almost immediately decreased (range 15–83%). In infants younger than three months the decrease was statistically significantly larger than older infants. The decrease in heart rate was often combined with vegetative responses, like flush.	Bradycardia (n = 279)	Short gentle thrust in suboccipital region (50 Newton), performed by a manual therapist	Observa-tional study	Koch et al., 2002 [66]	Moderate
Cohort of 199 infants	Muscle tension disorders of mouth or pharynx or asymmetry of skull, neck, trunk or hip	Responses after an upper cervical impulse were investigated. Physiological responses were shown in 53%; flush (49%), short spells of apnea (22%), hyperextension of the back and/or neck (13%) and sweating (8%). The short spells of apnea lasted less than 10 seconds and breathing pattern was immediately restored by blowing into the child’s face. The authors stated that these responses were normal physiological responses and cannot be interpreted as adverse reaction or harm.	Physiological responses (n = 105)	Short gentle thrust (50 Newton) in suboccipital region, performed by a manual therapist	Observa-tional study	Koch et al., 1998 [74]	Moderate
Cohort of 114 cases of infants (<12 weeks)	Sub-optimal breast-feeding	Data abstraction out of case series to describe circumstances, clinical features, role and treatment outcomes.	No harms	Low force spinal mobilization, performed by chiropractors	Retro-spective case series	Miller et al., 2009 [68]	Moderate
**Cervical mobilizations in children/adolescents**							
No studies							
**Full spine mobilizations in infants**							
21-day-old girl	Colic and fussiness	After manipulation infant immediately cried and fell asleep. Infant remained fussy and the mother felt a crackling sensation of the back. X-ray showed acute fractures of the 7^th^ and 8^th^ posterior left ribs. No additional fractures were found. Infant went for follow-up to the child abuse center. Results of bone laboratory tests were normal. The center concluded that child abuse could not be definitively ruled out, but chiropractic manipulation was seen as a plausible explanation for the fractures.	Rib fractures	Spinal fingertip pressure and adjustments using a ‘spring-activated device’, performed by a chiropractor	Case report	Wilson et al., 2012 [73]	High
Cohort of 194 infants	Various conditions	Data were extracted from mother’s completed questionnaires about infant characteristics, symptoms and perceived effect.	No harms	Low-force mobilizations of spinal joints in the area of dysfunction, performed by chiropractors	Cross-sectional survey	Nicolas-Schmid et al., 2016 [62]	High
Cohort of 104 infants (<4 weeks)	Colic	Infants were randomized to SMT and no treatment. Parents reported on adverse events during the treatment period.	No harms	Low-force spinal mobilization (2 Newton), performed by a chiropractor	RCT	Miller et al., 2012 [50]	Moderate
**Full spine mobilizations in children/adolescents**							
No studies							
**Unspecified treatment techniques**							
956 chiro-practors reported on treatment of children (0–18 years)	Various conditions	A survey was used to investigate characteristics of pediatric chiropractic practice, including side effects.	Unspecified mild and moderate harms (n = 557)	Treatment techniques were not specified. Treatments were performed by chiropractors	Cross-sectional survey	Marchand et al., 2012 [61]	Moderate

All observational studies and case reports showed methodological shortcomings and moderate-to-high risk of bias, suggesting a negative impact on the quality of evidence (see S3 Table). Studies lacked details about the performed treatment and information on the background, education/training and experience of professionals were often not provided.

#### Infants

Three case reports described adverse events in infants after cervical HVLA manipulations including death[71, 72] and temporary paralysis.[70] In all case reports, these adverse events could not be demonstrated to be a direct effect of cervical HVLA manipulations, rather, they were suspected to be related to missed underlying pathology. No studies reporting on harms after full spine HVLA manipulations were found.

One case report described a severe harm of rib fractures after mobilizations of the full spine using an Activator device in an infant. Physical abuse was suspected but could not be proved.[73] Two observational studies, including a total of 894 infants showed mild harms in terms of transient physiological responses and side effects, such as bradycardia and flush (n = 384), after short, gentle thrust cervical mobilizations.[66, 74] Three studies (n = 412) reported no harms occurred after spinal mobilizations; a retrospective case series (n = 114) reported no harms occurred after cervical mobilizations[68] and an observational study (n = 104) and a controlled study (n = 194) reported that no harms occurred after full spine mobilizations in infants.[50, 62]

#### Children/adolescents

Three studies described harms after cervical HVLA manipulation in children/adolescents. One case report described a severe harm of muscle weakness.[69] Two controlled studies reported mild, transient harms in terms of side effects: one study (n = 52) reported dizziness (n = 11) and hot skin (n = 26),[55] one study (n = 18) reported neck pain (n = 1) and headache (n = 1).[59] Five studies reported harms after HVLA manipulations performed on the full spine. In three of these studies (n = 1529) a small number of mild harms (n = 9) was reported;[60, 64, 67] the other two studies (n = 145) reported no harms.[53, 63] No studies were found reporting on harms after cervical or full spine mobilizations. One study (n = 956) reported side effects or reactions in children after chiropractic treatment (n = 557), but both side effects or reactions and treatment techniques were not specified.[61] Hence, conclusions on treatment technique cannot be given.

## Discussion

This review provides a unique overview of the evidence investigating the effectiveness and safety of specific SMT techniques specified per treatment indication and age group, instead of concluding on SMT as a general treatment approach. We found limited evidence for all age groups and treatment indications; overall the body of evidence is of very low quality due to moderate-to-high risk of bias, imprecise estimates, and lack of demonstrated consistency across studies. The effectiveness of gentle, low-velocity spinal mobilizations in infants with colic or torticollis remains uncertain. The effectiveness of HVLA spinal manipulations to manage asthma, nocturnal enuresis, headache, idiopathic scoliosis, and to improve grip strength in children and/or adolescents, also remains uncertain. We found that the number of reports of severe harms as direct side effects of SMT techniques were scarce and may be underreported. Where reported, harms differed between treatment techniques and between age groups. Gentle, low-velocity mobilization techniques appear to be a safe treatment technique in infants and children and/or adolescents. Cervical and full spine HVLA manipulations, however, might be associated with severe harms, although underlying pathology was suspected in the cases reported on.

### Effectiveness of SMT techniques

The very low quality of the body of evidence prevented us from drawing clinically meaningful conclusions on effectiveness of specific SMT techniques for specified treatment indications. These findings are consistent with previous reviews investigating the effectiveness of pediatric manual therapy as a general treatment approach.[1, 2, 4, 13] Specifically, the systematic review of Bronfort et al. (2010) also concluded that effectiveness of SMT in children is uncertain.[13] However, Bronfort et al. summarized the evidence regarding general manual treatment performed in both adults and children, and included various interventions, such as spinal and extremity joint manipulation or mobilization, craniosacral and osteopathic therapies and massage. In contrast to our systematic review, Bronfort et al. did not distinguish between SMT techniques in their analysis. Even though in our systematic review five additional (randomized) controlled studies were included, available literature was re-examined using the state-of-the-art GRADE methodology, and harms were examined in relation to specific treatment techniques, our conclusion about the lack of evidence remains largely the same as previous research. Our review sets itself apart from previously performed research by focusing on specific SMT treatment techniques, instead of making conclusions about SMT as a general therapeutic approach.

A large number of the included studies in our review showed shortcomings. We highlight these shortcomings here in an attempt to emphasize the need of high quality future research and reporting. First, authors reported a hypothesized relation between the child’s (non-)musculoskeletal condition and a particular spinal dysfunction.[49, 51–55, 57, 58] However, intermediate outcomes to assess or indicate this potential dysfunction, such as range of motion, were often neglected and only scarcely described. All studies assessed parent- or patient-reported outcomes to indicate perceived treatment effect, while only four out of twelve controlled studies additionally assessed functional outcomes to evaluate spinal dysfunction, such as change in torticollis,[52] lung function[53, 54] and grip strength.[59] Therefore, currently, no conclusions on the effect of specific SMT techniques on spinal dysfunction in these patients can be drawn. In future research it is important to include these intermediate outcomes in addition to patient-reported outcomes. Second, we would like to highlight that for adequate interpretation it is of great importance that studies provide a detailed description of the SMT technique performed. Important information regarding the specific treatment technique was often omitted from publications. As a consequence, it is challenging (or even impossible) for researchers and, maybe more importantly, healthcare professionals to interpret study findings and draw scientifically substantiated conclusions about effective treatment techniques. As such, this will hamper translation of study findings to clinical practice. Third, in the majority of the included controlled studies, decrease in complaints/symptoms and improvement in function over time was seen in both the intervention and control group. This may suggest a potentially favorable natural course for the indications under study. However, the majority of studies did not describe or consider this phenomenon. They focused on changes due to the intervention and only emphasized differences over time within the intervention group, instead of between group differences. Apart from a potential favorable natural course, the observed decrease in complaints/symptoms or improvement in function in the sham or control group may have occurred by other treatment effect, including placebo effect. To manage this, and to gain a better understanding of the course of complaints/symptoms over the longer time, effectiveness of SMT treatment techniques and potential harms of treatment, we recommend a change in study designs and a shift in the focus of research. We underline the importance of RCT designs using three-group-comparisons where a non-treatment group should be included. Moreover, we recommend research to focus on examining outcomes of specific SMT techniques and describing effectiveness in relation to these techniques, instead of making conclusions on SMT as a general treatment approach.

### Harms of SMT techniques

Worldwide, manual therapy is regularly performed in children of all ages. Previous reports indicate that 5 to 40% of patients receiving manual therapy are younger than 18 years old.[3, 9, 10, 27, 75–78] In view of this, severe harms such as death, paralysis and rib fractures after HVLA manipulations[69–72] or spinal instrumented-adjustments[73] are rare. Authors often concluded that underlying preexisting pathology was found and potentially related to the occurrence of these severe harms, and HVLA manipulations were not the direct cause of harm.[70, 72] Mild, transient harms, such as stiffness, soreness or headache, were reported in two controlled trials[55, 59], and five larger observational studies,[60, 61, 64, 67, 74] but may be underreported. Due to the lack of reported information on the specific treatment technique, specific symptoms and indications, and professional background of the health care professional, and because of the unknown total prevalence of pediatric SMT performed worldwide, conclusions about the prevalence of harms cannot be made and harms may be underreported. Taking these limitations into account, conclusions about the risk of harm and safety of SMT techniques are hard to draw. As such, we would encourage researchers to include detailed descriptions of specific performed techniques and details about the education and clinical experience of performing therapists. Moreover, to improve transparency and quantification of harms, we acknowledge the importance of continuous review of harms, as previously indicated by Vohra et al. and Humphreys et al.[79, 80] Observational cohorts with a longer follow up period could provide a more realistic estimation on risk of harm of a specific intervention in comparison to non-placebo controlled trials, in which strict inclusion criteria could limit the representation of a realistic study population.[32] Furthermore, databases and registries of performed treatments in infants and children could facilitate the reporting and review of harms. Such resources provide a mechanism to continuously monitor treatment outcomes and harms, and could be more reliable for reporting on harms as they do not aim to collect data for research in only a specific period and population.[32]

### Strengths and limitations

Our systematic review has a number of strengths. Our review sets itself apart from previous research by focusing on the effectiveness and harms of specific SMT treatment techniques, instead of concluding about SMT as a general therapeutic approach. A further strength is that we examined the evidence for infants separately from children and/or adolescents, providing a more nuanced overview of the effectiveness and safety of SMT techniques in children of different ages. In addition, we assessed the quality of the body of evidence using GRADE.

A limitation is that meta-analysis could only be performed for one comparison and on one outcome due to low consistency across studies. Sparse data meant that the quality of evidence for any given comparison of treatments and treatment outcome was very low. Finally, many studies were excluded from the review because they did not report on harms. Importantly, this does not necessarily indicate absence of harms and may underestimate the occurrence of harms.

## Conclusion

Due to very low quality of the evidence, the effectiveness of gentle, low-velocity mobilizations in infants and HVLA manipulations in children and/or adolescents is uncertain. Assessments of intermediate outcomes are lacking in current pediatric SMT research. Therefore, the relationship between specific treatment and its effect on the hypothesized spinal dysfunction remains unclear. Gentle, low-velocity spinal mobilizations seem to be a safe treatment technique. Although scarcely reported, HVLA manipulations in infants and young children could lead to severe harms. Severe harms were likely to be associated with unexamined or missed underlying medical pathology. Nevertheless, there is a need for high quality research to increase certainty about effectiveness and safety of specific SMT techniques in infants, children and adolescents. We encourage conduction of controlled studies that focus on the effectiveness of specific SMT techniques on spinal dysfunction, instead of concluding about SMT as a general treatment approach. Large observational studies could be conducted to monitor the course of complaints/symptoms in children and to gain a greater understanding of potential harms.

## Supporting information

S1 Table Inclusion and exclusion criteria. (DOCX)

S2 Table Excluded studies of the systematic review. (DOCX)

S3 Table Risk of bias tables. (DOCX)

S4 Table GRADE tables. (DOCX)

S1 File PRISMA checklist. (DOC)
