# Supplementary material for: Spinal manual therapy in infants, children and adolescents: A systematic review and meta-analysis on treatment indication, technique and outcomes
Source: PLoS One. 2019 Jun 25;14(6):e0218940. doi: 10.1371/journal.pone.0218940 (PMC6592551; doi:10.1371/journal.pone.0218940)
Supplement: S1 Table — (DOCX) [file pone.0218940.s001.docx]

**S1 Table. Inclusion and exclusion criteria**

| **Inclusion criteria** | **Exclusion criteria** |
| --- | --- |
| **Population:**  Children between 0 and 18 years | **Population:**  Mix of children and adults without stratification of the outcomes per age group |
| **Intervention:**  Spinal manual therapy as primary intervention | **Intervention:**  Manual therapies with the primary focus on manipulating fractures (orthopedics), multiple body parts, extremities, fasciae, soft tissue, nervous system. |
| **Outcomes:**  Any objective or subjective outcome reported by therapists, parents or children |  |
| **Language:**  Studies published in English, Dutch or German |  |
| **Study designs:**  ***Studies describing effectiveness*** | |
| (Randomized) controlled trials/studies |  |
| ***Studies describing harms or safety issues*** | |
| Observational studies: descriptive studies, retrospective and prospective cohort studies  Case series and/or case reports |  |
| Intervention studies in which harms are reported as secondary outcome |  |

Only unique studies were eligible for further screening; systematic reviews were not included, but studies embedded in these reviews were. A distinction in eligibility criteria was made between studies on effectiveness of SMT and studies reporting on harms. Only controlled studies were included to describe effectiveness. Harms described as secondary outcome in included controlled studies were also reported.

Harms are usually reported in observational, descriptive studies and case reports,[1] and observational studies are necessary for adequate assessment of harms, especially in vulnerable populations, such as children.[2] Therefore, observational study designs were only included if they reported on harms of SMT in infants, children and/or adolescents as primary or secondary study outcome.

1. Cochrane Effective Practice and Organisation of Care (EPOC). *Taking account of adverse effects in EPOC reviews.* EPOC Resources for review authors;2017.

2. Chou R, Aronson N, Atkins D, et al. AHRQ series paper 4: assessing harms when comparing medical interventions: AHRQ and the effective health-care program. *J Clin Epidemiol.* 2010;63(5):502-512.
