# Supplementary material for: Spinal manual therapy in infants, children and adolescents: A systematic review and meta-analysis on treatment indication, technique and outcomes
Source: PLoS One. 2019 Jun 25;14(6):e0218940. doi: 10.1371/journal.pone.0218940 (PMC6592551; doi:10.1371/journal.pone.0218940)
Supplement: S2 Table — (DOCX) [file pone.0218940.s002.docx]

**S2 Table. Excluded studies of the systematic review**

| **Excluded study (reference)** | **Reason for exclusion** |
| --- | --- |
| Accorsi, A., Lucci, C., Di Mattia, L., Granchelli, C., Barlafante, G., Fini, F., et al. (2014). Effect of osteopathic manipulative therapy in the attentive performance of children with attention-deficit/hyperactivity disorder. *J Am Osteopath Assoc, 114*(5), 374-381. | (Primary) intervention could not be described as SMT |
| Alcantara, J., & Davis, J. (2010). The chiropractic care of children with attention-deficit/hyperactivity disorder: a retrospective case series. *Explore (NY), 6*(3), 173-182. | Study population |
| Brady, N. S. (2010). Efficacy of a standardized osteopathic manipulative therapy protocol on pulmonary function and symptomatology in moderate persistent asthmatic children. *Annals of Allergy, Asthma and Immunology. Conference: 2010 Annual Meeting of the American College of Allergy, Asthma and Immunology Phoenix, Arizona United States. Conference Start: 20101111 Conference End: 20101116. Conference Publication: (var.pagings), 105*(5), A36 | Study design |
| Brassard, R. (2007). Adverse events in the manipulation of pediatric patients: flaws in a systematic review. *Pediatrics, 119*(6), 1265-1266; author reply 1266-1267. | Study design |
| Bronfort, G., Evans, R. L., Kubic, P., & Filkin, P. (2001). Chronic pediatric asthma and chiropractic spinal manipulation: a prospective clinical series and randomized clinical pilot study. *J Manipulative Physiol Ther, 24*(6), 369-377. | Outcomes |
| Chen, J., & Lantz, C. A. (2001). Effect of chiropractic intervention on small scoliotic curves in younger subjects: a time-series cohort design. *Abstract*. | Study design |
| Crowther, E. R., Feise, R. J., Grod, J. P., Menke, J. M., Rowe, D. E., Stoline, M. R., & Schaller, T. M. (2005). Chiropractic manipulation in adolescent idiopathic scoliosis: A pilot study. *Chiropr & Osteopat, 13*(1), Online access only 37 p. | Study population |
| Cuthbert, S. C., & Barras, M. (2009). Developmental delay syndromes: psychometric testing before and after chiropractic treatment of 157 children. *J Manipulative Physiol Ther, 32*(8), 660-669. | (Primary) intervention could not be described as SMT |
| Davies, N. J. (2002). Chiropractic management of deformational plagiocephaly in infants: An alternative to device-dependent therapy. *Chiropr J Aust, 32*(2), 52-55. | Study design |
| Degenhardt, B. F., & Kuchera, M. L. (2006). Osteopathic evaluation and manipulative treatment in reducing the morbidity of otitis media: a pilot study. *J Am Osteopath Assoc, 106*(6), 327-334. | (Primary) intervention could not be described as SMT |
| Elster, E. (2009). Sixteen infants with acid reflux and colic undergoing upper cervical chiropractic care to correct vertebral subluxation: A retrospective analysis of outcome [case report]. *J Pediatr Matern & Fam Health - Chiropr, 2009*(2), Online access 7 p. | Outcomes |
| Ernst, E. (2002). Manipulation of the cervical spine: a systematic review of case reports of serious adverse events, 1995 - 2001 (Structured abstract). *Medical Journal of Australia, 176*(8), 376-380 | Study design |
| Froehle, R. M. (1996). Ear infection: a retrospective study examining improvement from chiropractic care and analyzing for influencing factors. *J Manipulative Physiol Ther, 19*(3), 169 | Outcomes |
| Giesen, J. M., Center, D. B., & Leach, R. A. (1989). An evaluation of chiropractic manipulation as a treatment of hyperactivity in children. *J Manipulative Physiol Ther, 12*(5), 353 | Study population |
| Graham, R. L., & Pistolese, R. A. (1997). An impairment rating analysis of asthmatic children under chiropractic care. *J Vert Sublux Res, 1*(4), p. 1 | Outcomes |
| Guiney, P. A., Chou, R., Vianna, A., & Lovenheim, J. (2005). Effects of osteopathic manipulative treatment on pediatric patients with asthma: a randomized controlled trial. *J Am Osteopath Assoc, 105*(1), 7 | (Primary) intervention could not be described as SMT |
| Jaszewski, E., Harden, J., & Smith, M. (2016). Improvement in idiopathic scoliotic and sub-scoliotic curvatures in children following subluxation correction utilizing the Pierce Results System: A retrospective analysis of outcomes. *J Pediatr Matern & Fam Health - Chiropr, 2016*(4), Online access only p 118-126. | Study design |
| Kukurin, G. W. (2002). Chronic pediatric asthma and chiropractic spinal manipulation: a prospective clinical series and randomized clinical pilot study. *J Manipulative Physiol Ther, 25*(8), 540 | Study design |
| Langkau, J., & Miller, J. (2012). An investigation of musculoskeletal dysfunctions in infants including a case series of KISS-diagnosed children. *J Clin Chiropr Pediatr, 13*(1), 958-967. | Study design |
| Lantz, C. A., & Chen, J. (2001). Effect of chiropractic intervention on small scoliotic curves in younger subjects: a time-series cohort design. *J Manipulative Physiol Ther, 24*(6), 385-393. | Outcomes |
| Marchand, A. M. (2015). A Proposed Model With Possible Implications for Safety and Technique Adaptations for Chiropractic Spinal Manipulative Therapy for Infants and Children. *J Manipulative Physiol Ther, 38*(9), 713 | Outcomes |
| Marchand, A. M., Miller, J. E., & Mitchell, C. (2009). Diagnosis and chiropractic treatment of infant headache based on behavioral presentation and physical findings: a retrospective series of 13 cases. *J Manipulative Physiol Ther, 32*(8), 682 | Outcomes |
| Martelli, M., Cardinali, L., Barlafante, G., Pizzolorusso, G., Renzetti, C., & Cerritelli, F. (2014). Do placebo effects associated with sham osteopathic procedure occur in newborns? Results of a randomized controlled trial. *Complement Ther Med, 22*(2), 197 | (Primary) intervention could not be described as SMT |
| Miller, J., Beharie, M. C., Taylor, A. M., Simmenes, E. B., & Way, S. (2016). Parent Reports of Exclusive Breastfeeding After Attending a Combined Midwifery and Chiropractic Feeding Clinic in the United Kingdom: A Cross-Sectional Service Evaluation. *J Evid Based Complementary Altern Med, 21*(2), 85-91. | Study design |
| Miller, J.; Newell, D.; Bolton, J. (2010). Chiropractic manual therapy for the infant with colic crying: a randomised double blind placebo-controlled trial. *Clin Chiropract,* 13(2), 178-80 | Study design |
| Miller, J., & Klemsdal, M. (2008). Can chiropractic care improve infants' sleep? *J Clin Chiropr Pediatr, 9*(1), 543 | Outcomes |
| Navrud, I. M., Miller, J., Bjørnli, M. E., Feier, C. H., & Haugse, T. (2014). A survey of parent satisfaction with chiropractic care of the pediatric patient. *J Clin Chiropr Pediatr, 14*(3), Online access only p 1167 | Study population |
| O'Neal, M. L. (2003). The pediatric spine: anatomical and dynamic considerations preceding manipulation. *Compr Ther, 29*(2-3), 124-129. | Study design |
| Pistolese, R. A. (1998). Risk assessment of neurological and/or vertebrobasilar complications in the pediatric chiropractic patient. *J Vert Sublux Res, 2*(2), p. 1-9. | Study design |
| Pohlman, K. A., Carroll, L., Hartling, L., Tsuyuki, R., & Vohra, S. (2016). Attitudes and opinions of doctors of chiropractic specializing in pediatric care toward patient safety: A cross-sectional survey. *J Manipulative Physiol Ther, 39*(7), 487-493. | Study design |
| Pohlman, K. A., Carroll, L., Hartling, L., Tsuyuki, R. T., & Vohra, S. (2016). Barriers to Implementing a Reporting and Learning Patient Safety System: Pediatric Chiropractic Perspective. *J Evid Based Complementary Altern Med, 21*(2), 105-109. | Study design |
| Raith, W., Marschik, P. B., Sommer, C., Maurer-Fellbaum, U., Amhofer, C., Avian, A., et al. (2016). General Movements in preterm infants undergoing craniosacral therapy: a randomised controlled pilot-trial. *BMC Complement Altern Med, 16*, 12. | (Primary) intervention could not be described as SMT |
| Rowe, D. E., Feise, R. J., Crowther, E. R., Grod, J. P., Menke, J. M., Goldsmith, C. H., et al. (2006). Chiropractic manipulation in adolescent idiopathic scoliosis: a pilot study. *Chiropr Osteopat, 14*, 15. | Study population |
| Schulz, C., Leininger, B., Evans, R., Vavrek, D., Peterson, D., Haas, M., & Bronfort, G. (2014). Spinal manipulation and exercise for low back pain in adolescents: Study protocol for a randomized controlled trial. *Chiropr Man Therap, 22*(1). | Study design |
| Steele, K. M., Carreiro, J. E., Viola, J. H., Conte, J. A., & Ridpath, L. C. (2014). Effect of osteopathic manipulative treatment on middle ear effusion following acute otitis media in young children: a pilot study. *J Am Osteopath Assoc, 114*(6), 436-447. | (Primary) intervention could not be described as SMT |
| Stewart, A. (2012). Paediatric chiropractic and infant breastfeeding difficulties: A pilot case series study involving 19 cases. *Chiropr J Aust, 42*(3), 98-107. | Study design |
| Vallone, S. A., Fysh, P. N., & Tanis, L. (2009). First do no harm: Chiropractic care and the newborn. *J Clin Chiropr Pediatr, 10*(2), 647-654. | Study design |
| van Poecke, A. J., & Cunliffe, C. (2009). Chiropractic treatment for primary nocturnal enuresis: a case series of 33 consecutive patients. *J Manipulative Physiol Ther, 32*(8), 675-681. | (Primary) intervention could not be described as SMT |
