# Supplementary material for: Spinal manual therapy in infants, children and adolescents: A systematic review and meta-analysis on treatment indication, technique and outcomes
Source: PLoS One. 2019 Jun 25;14(6):e0218940. doi: 10.1371/journal.pone.0218940 (PMC6592551; doi:10.1371/journal.pone.0218940)
Supplement: S3 Table — (DOCX) [file pone.0218940.s003.docx]

**S3 Tables. Risk of bias tables**

**Table A. Risk of bias of controlled studies using the Cochrane Risk of Bias tool**

| **Study** | **Q1** | **Q2** | **Q3** | **Q4**  **Parent or**  **patient**  **reported** | **Q4**  **Level of**  **dysfunction** | **Q5**  **Parent or**  **patient**  **reported** | **Q5**  **Level of**  **dysfunction** | **Q6** | **Q7** |
| --- | --- | --- | --- | --- | --- | --- | --- | --- | --- |
| **Balon et al.,**  **1998^3^** |  |  |  |  |  |  |  |  |  |
| **Borusiak et al.,**  **2009^4^** |  |  |  |  |  |  |  |  |  |
| **Botelho &**  **Andrade, 2012^5^** |  |  |  |  |  |  |  |  |  |
| **Browning &**  **Miller, 2008^6^** |  |  |  |  |  |  |  |  |  |
| **Haugen et al.,**  **2010^7^** |  |  |  |  |  |  |  |  |  |
| **Khorshid et al.,**  **2006^8^** |  |  |  |  |  |  |  |  |  |
| **Miller et al.,**  **2012^9^** |  |  |  |  |  |  |  |  |  |
| **Olafsdottir et al.,**  **2001^10^** |  |  |  |  |  |  |  |  |  |
| **Reed et al.,**  **1994^11^** |  |  |  |  |  |  |  |  |  |
| **Wiberg et al.,**  **1999^12^** |  |  |  |  |  |  |  |  |  |

*unclear: description is too unclear to give consideration.

Q1: Random sequence generation (selection bias); Q2: Allocation concealment (selection bias); Q3: Blinding of participants and personnel (performance bias); Q4: Blinding of outcome assessment (detection bias; per outcome); Q5: Incomplete outcome data (attrition bias; per outcome); Q6: Selective reporting (reporting bias); Q7: Other bias (e.g. no between group comparison).

|  | **No risk of bias** |
| --- | --- |
|  | **Risk of bias** |
|  | **Not applicable** |
|  | **Unclear*** |

**Table B. Risk of bias in observational and descriptive studies using the RTI Item Bank for observational studies**

| **Study** | **Q1** | **Q2** | **Q3** | **Q4** | **Q5** | **Q6** | **Q7** | **Q8** | **Q9** | **Q10** | **Q11** | **Q12** | **Q13** |
| --- | --- | --- | --- | --- | --- | --- | --- | --- | --- | --- | --- | --- | --- |
| **Cohort studies** | | | | | | | | | | | | | |
| **Hayden et al., 2003^13^** |  |  |  |  |  |  |  |  |  |  |  |  |  |
| **Miller & Benfield, 2008^14^** |  |  |  |  |  |  |  |  |  |  |  |  |  |
| **Nicolas-Schmid et al., 2016^15^** |  |  |  |  |  |  |  |  |  |  |  |  |  |
| **LeBoeuf et al., 1991^16^** |  |  |  |  |  |  |  |  |  |  |  |  |  |
| **Case series** | | | | | | | | | | | | | |
| **Miller et al., 2009^17^** |  |  |  |  |  |  |  |  |  |  |  |  |  |
| **Cross-sectional studies** | | | | | | | | | | | | | |
| **Alcantara et al., 2009^18^** |  |  |  |  |  |  |  |  |  |  |  |  |  |
| **Koch et al., 1998^19^** |  |  |  |  |  |  |  |  |  |  |  |  |  |
| **Koch et al., 2002^20^** |  |  |  |  |  |  |  |  |  |  |  |  |  |
| **Marchand et al., 2012^21^** |  |  |  |  |  |  |  |  |  |  |  |  |  |

*not applicable because of study design (e.g. no comparison group). Q1: Do the inclusion/exclusion criteria vary across the comparison groups of the study? Q2: Does the strategy for recruiting participants into the study differ across groups? Q3: Is the selection of the comparison group inappropriate, after taking into account feasibility and ethical considerations? Q4: Does the study fail to account for variations in the execution of the study from the proposed protocol? Q5: Was the outcome assessor not blinded to the intervention or exposure status of participants? Q6: Were valid and reliable measures, implemented consistently across all study participants used to assess inclusion/exclusion criteria, intervention/exposure outcomes, participant health benefits and harms, and confounding? Q7: Was the length of follow up different across study groups? Q8: In cases of high loss to follow up, was the impact assessed? Q9: Are any important primary outcomes missing from the results? Q10: Are any important harms of adverse events that may be a consequence of the intervention/exposure missing from the results? Q11: Are results believable considering study limitations? Q12: Any attempt to balance the allocation between groups or match groups? Q13: Were important confounding variables taken into account in the design and/or analysis?

|  | **No risk of bias** |
| --- | --- |
|  | **Risk of bias** |
|  | **Partial risk of bias** |
|  | **Not applicable*** |

**Table C. Risk of bias in case reports using the JBI Critical Appraisal Checklist for Case Reports**

| **Study** | **Q1** | **Q2** | **Q3** | **Q4** | **Q5** | **Q6** | **Q7** | **Q8** |
| --- | --- | --- | --- | --- | --- | --- | --- | --- |
| **Deputy, 2004^22^** |  |  |  |  |  |  |  |  |
| **Shafrir & Kaufman, 1996^23^** |  |  |  |  |  |  |  |  |
| **Holla et al., 2009^24^** |  |  |  |  |  |  |  |  |
| **Jacobi et al., 2001^25^** |  |  |  |  |  |  |  |  |
| **Wilson et al., 2012^26^** |  |  |  |  |  |  |  |  |

Q1: Were patient’s demographic characteristics clearly described? Q2: Was the patient’s history clearly described and presented as a timeline? Q3: Was the current clinical condition of the patient on presentation clearly described? Q4: Were diagnostic tests or assessment methods and the results clearly described? Q5: Was the intervention or treatment procedure clearly described? Q6: Was the post-intervention clinical condition clearly described? Q7: Were adverse events (harms) or unanticipated events identified and described? Q8: Does the case report provide takeaway lessons?

|  | **No risk of bias** |
| --- | --- |
|  | **Risk of bias** |
|  | **Partial risk of bias** |
