# Supplementary material for: Spinal manual therapy in infants, children and adolescents: A systematic review and meta-analysis on treatment indication, technique and outcomes
Source: PLoS One. 2019 Jun 25;14(6):e0218940. doi: 10.1371/journal.pone.0218940 (PMC6592551; doi:10.1371/journal.pone.0218940)
Supplement: S4 Table — (DOCX) [file pone.0218940.s004.docx]

**S4 Tables. GRADE tables**

| **Author(s):** Femke Driehuis, Thomas J. Hoogeboom, Maria W.G. Nijhuis – van der Sanden, Rob A. de Bie, J. Bart Staal  **Date:** 10-01-2018  **Question:** Is manual therapy more effective than no treatment in reducing crying hours in infants with colic?  **Settings:** primary manual therapy care  **Bibliography:** Olafsdottir et al., 2001; Miller et al., 2012 | | | | | | | | | | |
| --- | --- | --- | --- | --- | --- | --- | --- | --- | --- | --- |
| **Quality assessment** | | | | | | | **Summary of findings** | | | |
| **No of studies** | **Design** | **Risk of bias** | **Inconsistency** | **Indirectness** | **Imprecision** | **Publication bias** | **Intervention (n)** | **Control**  **(n)** | **Standardized mean difference**  **(95% CI)** | **Quality** |
| **Outcome: crying hours per day** | | | | | | | | | |  |
| 2 | Randomized controlled trials | Serious (1) | Very serious (2) | Serious (3) | Serious (4) | Undetected | 114* | 74 | -0.33 (-0.12; 0.59) | Very low |

IV: intervention group, C: control group, *Miller et al., 2012 included two intervention groups. According to the Cochrane Handbook intervention groups were combined and mean and SD were calculated.

1: moderate risk of bias. Downgrade one level.

2: inconsistency in findings.

3: population, intervention, outcomes are similar.

4: sample size is not big enough to detect precise estimate of effect (less than 400) and wide 95% CI’s.

| **Author(s):** Femke Driehuis, Thomas J. Hoogeboom, Maria W.G. Nijhuis – van der Sanden, Rob A. de Bie, J. Bart Staal  **Date:** 10-01-2018  **Question:** Is manual therapy more effective than other treatments in reducing crying hours in infants with colic?  **Settings:** primary manual therapy care  **Bibliography:** Browning & Miller, 2008; Wiberg et al., 1999 | | | | | | | | | | | |
| --- | --- | --- | --- | --- | --- | --- | --- | --- | --- | --- | --- |
| **Quality assessment** | | | | | | | **Summary of findings** | | | | |
| **No of studies** | **Design** | **Risk of bias** | **Inconsistency** | **Indirectness** | **Imprecision** | **Publication bias** | **Study** | **IV (n)** | **C (n)** | **Impact**  **(mean (SD))** | **Quality** |
| **Outcome: crying hours per day** | | | | | | | | | | |  |
| 2 | Randomized controlled trials | Serious (1) | Very serious (2) | Serious (3) | Serious (4) | Undetected | Browning & Miller, 2008  Wiberg et al., 1999 | 22  25 | 21  25 | IV: -2.1 (2.2)  C: -2.0 (1.4)  IV: -2.4 (0.4)  C: -1.0 (0.6) | Very low |

IV: intervention group, C: control group

1: moderate risk of bias. Downgrade one level.

2: studies show inconsistency in outcomes; significant and non-significant differences in improvement between groups.

3: population, intervention, outcomes are similar. Comparison groups received different care, which is not routine care. Downgrade one level.

4: sample size is not big enough to detect precise estimate of effect (less than 400) and no or wide 95% CI’s reported.

| **Author(s):** Femke Driehuis, Thomas J. Hoogeboom, Maria W.G. Nijhuis – van der Sanden, Rob A. de Bie, J. Bart Staal  **Date:** 10-01-2018  **Question:** Is physical therapy combined with manual therapy for 8 weeks more effective than physical therapy alone in infants with torticollis?  **Settings:** primary manual therapy care  **Bibliography:** Haugen et al., 2010 | | | | | | | | | | |
| --- | --- | --- | --- | --- | --- | --- | --- | --- | --- | --- |
| **Quality assessment** | | | | | | | **Summary of findings** | | | |
| **No of studies** | **Design** | **Risk of bias** | **Inconsistency** | **Indirectness** | **Imprecision** | **Publication bias** | **Intervention (n)** | **Control (n)** | **Impact** | **Quality** |
| **Outcome: change in torticollis** | | | | | | | | | | |
| 1 | Randomized controlled trial | Not serious (1) | Unknown (2) | Not serious (3) | Very serious (4) | Undetected | 16 | 16 | IV: 80.0% improved  C: 81.3% improved | Very low |

IV: intervention group, C: control group

1: low risk of bias.

2: only one study. Inconsistency unknown.

3: population, intervention, outcomes are similar.

4: No 95%CI are provided. Sample size is very small. Too small to detect precise estimate of effect (less than 400)

| **Author(s):** Femke Driehuis, Thomas J. Hoogeboom, Maria W.G. Nijhuis – van der Sanden, Rob A. de Bie, J. Bart Staal  **Date:** 10-01-2018  **Question:** Is manual therapy more effective than sham treatment in children (6-17 years) with asthma on longer term (12-16 weeks)?  **Settings:** primary manual therapy care  **Bibliography:** Balon et al., 1998; Bronfort et al., 2002 | | | | | | | | | | | |
| --- | --- | --- | --- | --- | --- | --- | --- | --- | --- | --- | --- |
| **Quality assessment** | | | | | | | **Summary of findings** | | | | |
| **No of studies** | **Design** | **Risk of bias** | **Inconsistency** | **Indirectness** | **Imprecision** | **Publication bias** | **Study** | **IV (n)** | **C (n)** | **Impact**  **(mean (SD))** | **Quality** |
| **Outcome: lung function (FEV^1^, percentage improvement (%))** | | | | | | | | | | |  |
| 2 | Randomized controlled trials | Serious (1) | Serious (2) | Not serious (3) | Very serious (4) | Undetected | Balon et al., 1998  Bronfort et al., 2002 | 38  24 | 42  12 | IV: 103.6% (13.7)  C: 104.3% (13.3)  NA* | Very low |

IV: intervention group, C: control group, *NA: not applicable: outcomes control group were not reported

1: moderate risk of bias.

2: studies show inconsistency in magnitude of effect on lung function

3: population, intervention, outcomes are similar. Control outcomes and comparison care are similar

4: Wide 95% CI on lung function. No 95%CI’s on asthma related symptoms in both studies. Sample size is very small. Too small to detect precise estimate of effect (less than 400).

| **Author(s):** Femke Driehuis, Thomas J. Hoogeboom, Maria W.G. Nijhuis – van der Sanden, Rob A. de Bie, J. Bart Staal  **Date:** 10-01-2018  **Question:** Is upper cervical manual therapy more effective than full spine manual therapy in children with autism on longer term (3 months)?  **Settings:** primary manual therapy care  **Bibliography:** Khorsid et al., 2006 | | | | | | | | | | |
| --- | --- | --- | --- | --- | --- | --- | --- | --- | --- | --- |
| **Quality assessment** | | | | | | | **Summary of findings** | | | |
| **No of studies** | **Design** | **Risk of bias** | **Inconsistency** | **Indirectness** | **Imprecision** | **Publication bias** | **Intervention (n)** | **Control (n)** | **Impact**  **(mean (SD))** | **Quality** |
| **Outcome: improvement in autism related symptoms (%)** | | | | | | | | | | |
| 1 | Randomized controlled trial | Serious (1) | Unknown (2) | Not serious (3) | Very serious (4) | Undetected | 7 | 7 | IV: 32% (na)  C: 19% (na) | Very low |

IV: intervention group, C: control group, na: not applicable: standard deviations were not reported

1: high risk of bias

2: only one study. Inconsistency is unknown.

3: population, intervention, outcomes are similar.

4: No 95%CI are provided. Sample size is very small. Too small to detect precise estimate of effect (less than 400).

| **Author(s):** Femke Driehuis, Thomas J. Hoogeboom, Maria W.G. Nijhuis – van der Sanden, Rob A. de Bie, J. Bart Staal  **Date:** 10-01-2018  **Question:** Is spinal manual therapy for two months more effective than placebo treatment in children with headache?  **Settings:** primary manual therapy care  **Bibliography:** Borusiak et al., 2009 | | | | | | | | | | |
| --- | --- | --- | --- | --- | --- | --- | --- | --- | --- | --- |
| **Quality assessment** | | | | | | | **Summary of findings** | | | |
| **No of studies** | **Design** | **Risk of bias** | **Inconsistency** | **Indirectness** | **Imprecision** | **Publication bias** | **Intervention (n)** | **Control (n)** | **Impact**  **(mean (SD))** | **Quality** |
| **Outcome: percentage days with headache** | | | | | | | | | | |
| 1 | Randomized controlled trial | Not serious (1) | Unknown (2) | Not serious (3) | Very serious (4) | Undetected | 28 | 28 | IV: -9.7% (na)  C: -9.4% (na) | Very low |
| **Outcome: duration of headache (hours)** | | | | | | | | | | |
| 1 | Randomized controlled trial | Not serious (1) | Unknown (2) | Not serious (3) | Very serious (4) | Undetected | 28 | 28 | IV: -7.5 (na)  C: -6.6 (na) | Very low |
| **Outcome: intensity of headache (VAS)** | | | | | | | | | | |
| 1 | Randomized controlled trial | Not serious (1) | Unknown (2) | Not serious (3) | Very serious (4) | Undetected | 28 | 28 | IV: -0.3 (na)  C: 0.1 (na) | Very low |

IV: intervention group, C: control group, na: not applicable: standard deviations were not reported

1: low risk of bias

2: Single study; inconsistency is unknown.

3: population, intervention, outcomes are similar.

4: No 95% CI reported. Sample size is very small. Too small to detect precise estimate of effect (less than 400).

| **Author(s):** Femke Driehuis, Thomas J. Hoogeboom, Maria W.G. Nijhuis – van der Sanden, Rob A. de Bie, J. Bart Staal  **Date:** 10-01-2018  **Question:** Is HVLA manual therapy more effective than sham treatment in children (5-13 years) with nocturnal enuresis after 10 weeks?  **Settings:** primary manual therapy care  **Bibliography:** Reed et al., 1994 | | | | | | | | | | |
| --- | --- | --- | --- | --- | --- | --- | --- | --- | --- | --- |
| **Quality assessment** | | | | | | | **Summary of findings** | | | |
| **No of studies** | **Design** | **Risk of bias** | **Inconsistency** | **Indirectness** | **Imprecision** | **Publication bias** | **Intervention (n)** | **Control (n)** | **Impact**  **(mean (SD))** | **Quality** |
| **Outcome: frequency of bed wetting** | | | | | | | | | | |
| 1 | Randomized controlled trial | Serious (1) | Unknown (2) | Not serious (3) | Very serious (4) | Undetected | 31 | 15 | IV: -1.2% (2.2)  C: 17.9% (46.1%) | Very low |

IV: intervention group, C: control group

1: moderate risk of bias

2: Single study; inconsistency is unknown.

3: population, intervention, outcomes are similar.

4: No 95% CI reported. Ranges in outcomes were very wide, including participants who decreased and increased frequency of bed wetting. Sample size is very small. Too small to detect precise estimate of effect (less than 400).

| **Author(s):** Femke Driehuis, Thomas J. Hoogeboom, Maria W.G. Nijhuis – van der Sanden, Rob A. de Bie, J. Bart Staal  **Date:** 10-01-2018  **Question:** Is manual therapy more effective than no treatment in adolescents (15-18 years) with idiopathic scoliosis on the short term (3 weeks)?  **Settings:** primary manual therapy care  **Bibliography:** Swierkosz & Nowak, 2015 | | | | | | | | | | |
| --- | --- | --- | --- | --- | --- | --- | --- | --- | --- | --- |
| **Quality assessment** | | | | | | | **Summary of findings** | | | |
| **No of studies** | **Design** | **Risk of bias** | **Inconsistency** | **Indirectness** | **Imprecision** | **Publication bias** | **Intervention (n)** | **Control (n)** | **Impact**  **(mean (SD))** | **Quality** |
| **Outcome: quality of life (somatic & psychological)** | | | | | | | | | | |
| 1 | Clinical trial | Serious (1) | Unknown (2) | Not serious (3) | Very serious (4) | Undetected | 21 | 11 | Not applicable* | Very low |

*Outcomes control group not reported

1: Moderate risk of bias

2: Single study; inconsistency is unknown.

3: population, intervention, outcomes are similar.

4: No 95% CI reported. Clinical and control groups were not compared after treatment. Sample size is very small. Too small to detect precise estimate of effect (less than 400).

| **Author(s):** Femke Driehuis, Thomas J. Hoogeboom, Maria W.G. Nijhuis – van der Sanden, Rob A. de Bie, J. Bart Staal  **Date:** 10-01-2018  **Question:** Is HVLA manual therapy more effective than sham treatment in adolescent judo athletes in increasing grip strength directly after treatment?  **Settings:** primary manual therapy care  **Bibliography:** Botelho & Andrade, 2012 | | | | | | | | | | |
| --- | --- | --- | --- | --- | --- | --- | --- | --- | --- | --- |
| **Quality assessment** | | | | | | | **Summary of findings** | | | |
|  | | | | | | | **No of patients Effect** | | | |
| **No of studies** | **Design** | **Risk of bias** | **Inconsistency** | **Indirectness** | **Imprecision** | **Publication bias** | **Intervention** | **Control** | **Impact**  **(mean (SD))** | **Quality** |
| **Outcome: grip strength right hand** | | | | | | | | | | |
| 1 | Randomized controlled trial | Serious (1) | Unknown (2) | Not serious (3) | Very serious (4) | Undetected | 9 | 9 | IV: 10.5% (na)  C: 5.7% (na) | Very low |
| **Outcome: grip strength left hand** | | | | | | | | | | |
| 1 | Randomized controlled trial | Serious (1) | Unknown (2) | Not serious (3) | Very serious (4) | Undetected | 9 | 9 | IV: 16.8% (na)  C: 4.3% (na) | Very low |

IV: intervention group, C: control group, na: not applicable: standard deviations were not reported

1: High risk of bias

2: Single study; inconsistency is unknown.

3: population, intervention, outcomes are similar.

4: No 95% CI reported. Clinical and control groups were not compared after treatment. Sample size is very small. Too small to detect precise estimate of effect (less than 400)
